# Supplementary material for: Nematicidal Efficacy of a dsRNA-Chitosan Formulation Against Acrobeloides nanus Estimated by a Soil Drenching Application
Source: Biology (Basel). 2025 Sep 1;14(9):1161. doi: 10.3390/biology14091161 (PMC12467938; doi:10.3390/biology14091161)
Supplement: Supplementary file 1 [file biology-14-01161-s001.zip › biology-3775459-supplementary.pdf]

Supplementary Information

Table S1. Primer sequences used in this study

Table S2. GenBank accession numbers of ITS genes used to construct a phylogeny tree in Fig 2B

Table S3. Median lethal doses of three different dsRNAs in a microplate assay in Fig. 5A

Figure S1. Molecular identification and phylogenetic analysis of a nematode isolate using ITS sequence data. Nucleotide sequence of the internal transcribed spacer (ITS) region of a nematode isolate. The sequence was analyzed and color-coded to indicate different putative rDNA regions: partial 18S rDNA (yellow), ITS-1 (green), 5.8S rDNA (cyan), ITS-2 (magenta), and partial 28S rDNA (blue). These regions were identified based on sequence comparison with ITS sequences of *Heterorhabditis* spp. [49]

Figure S2. DNA sequences of *A. nanus* to produce the dsRNAs used in this study. Open reading frames of *Pat-10* (A), *Unc-87* (B), and *vATPase-B* (C) genes. The yellow-colored sequences represent the regions encoding their dsRNAs.

Table S1. Primer sequences used in this study

Genes	Sequence (5'-3')	Uses	Annealing temp (^0C)	Expected size (bp)
An-Unc-87	CAATCGACGTGAGACCACCA<br>GAAGACGGACCATGCCTTGA	RT-PCR<br>RT-qPCR	54.0	212
An-vATPase-B	AGAGGAAATGATCCAGACCGG<br>GCAGTCTCCATGTTGACTCCC	RT-PCR<br>RT-qPCR	55.0	219
An-Pat-10	ATACAAAAAGAGGAAGAC<br>ACGGCAGTGGCAAGATT	RT-PCR<br>RT-qPCR	57.0	118
T7+ An-Unc-87	<u>TAATACGACTCACTATAGGGAGA</u>CAATCGACGTGAGACCACCA<br><u>TAATACGACTCACTATAGGGAGA</u>GAAGACGGACCATGCCTTGA	RNAi	54.0	258
T7+An-vATPase-B	<u>TAATACGACTCACTATAGGGAGAAGAGGAAATGATCCAGACCGG</u><br><u>TAATACGACTCACTATAGGGAGAGCAGTCTCCATGTTGACTCCC</u>	RNAi	55.0	255
T7+ Pat-10	<u>TAATACGACTCACTATAGGGAGAATACAAAAAGAGGAAGAC</u><br><u>TAATACGACTCACTATAGGGAGAACGGCAGTGGCAAGATT</u>	RNAi	57.0	164
An-vATPase-B	Antisense-[FAM]GCAGTCTCCATGTTGACTCCC<br>Sense-[FAM]GGGAGTCAACATGGAGACTGC	FISH	-	-
Nematode ITS	TCCGTAGGTGAACCTGCGG<br>TCCTCCGCTTATTGATATGC	RT-PCR	50.0	808
An-Elongation Factor 1α	ATGGGTAAGGARAAGACACCAAG<br>CAGTACGCCCTGGAGGACAC	RT-PCR<br>RT-qPCR	54.0	173

Table S2. GenBank accession numbers of ITS genes used to construct a phylogeny tree in Fig 2B

organism name	GenBank accession number	Gene name	Order
Meloidogyne arenaria	MK188477.1	18S ribosomal RNA	Meloidogynidae
	MK188474.1	18S ribosomal RNA	
Meloidogyne javanica	JQ917440.1	18S ribosomal RNA	
	AF510060.1	5.8S ribosomal RNA	
Meloidogyne hapla	MK188479.1	18S ribosomal RNA	
	MK188472.1	18S ribosomal RNA	
Pratylenchus goodeyi	KF856291.1	18S ribosomal RNA	Pratylenchidae
	KF840456.1	18S ribosomal RNA	
	KM874803.1	18S ribosomal RNA	
Acrobeloides nanus	KY828308.1	18S ribosomal RNA	Cephalobidae
Ascaris lumbricoides	OQ539680.1	5.8S ribosomal RNA	Ascarididae
Toxocara cati	JF837173.1	5.8S ribosomal RNA	
	OK668292.1	5.8S ribosomal RNA	
Steinernema minutum	KY807715.1	5.8S ribosomal RNA	Steinernematidae
Steinernema carpocapsae	KC571265.1	18S ribosomal RNA	
	GQ421606.1	18S ribosomal RNA	
Oscheius myriophilus	OR606786.1	5.8S ribosomal RNA	Rhabditidae
	OR606787.1	5.8S ribosomal RNA	
Oscheius onirici	OP376147.1	5.8S ribosomal RNA	
	OP476677.1	5.8S ribosomal RNA	
Oscheius tipulae	OP476678.1	5.8S ribosomal RNA	
	OM480717.1	5.8S ribosomal RNA	

Table S3. Median lethal doses of three different dsRNAs in an agar plate assay in Fig. 5A

dsRNA	LC_{50} (95% CI), ppm	X^2	df	R^2
vATPase-B	84.2 (66.1~104.2)	50.13	1	0.8057
Pat-10	142.3 (110.2~186.8)	51.79	1	0.8298
Unc-87	283.6 (220.5~391.5)	72.03	1	0.7338

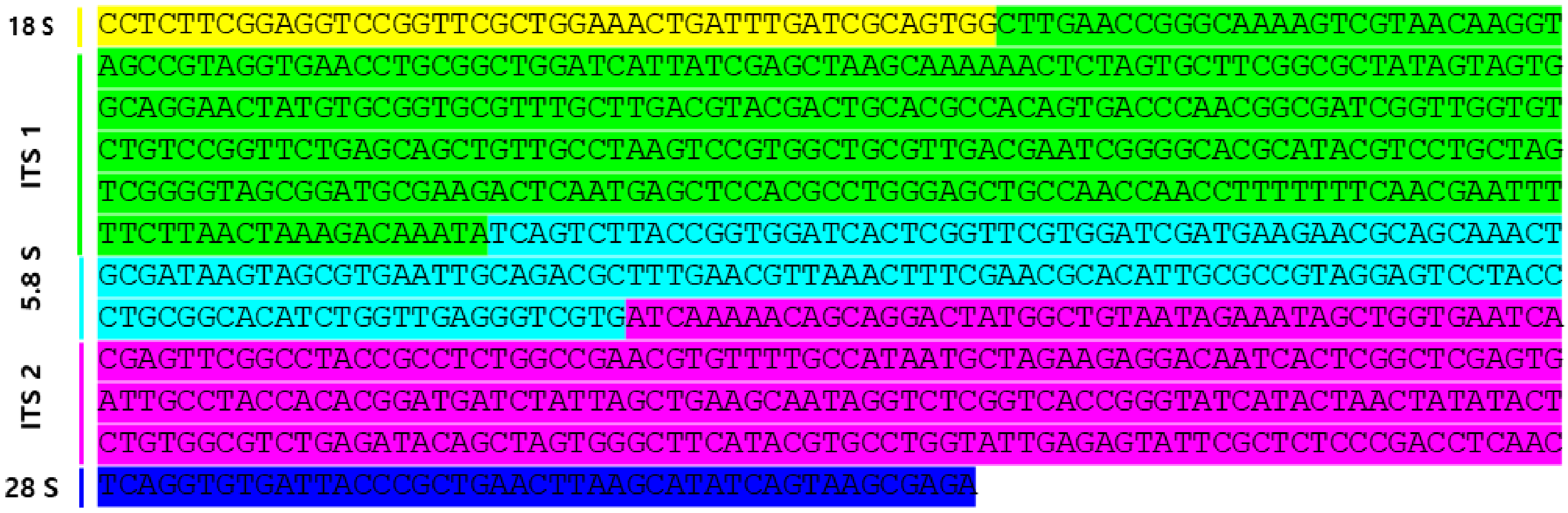
18 S
ITS 1
5.8 S
ITS 2
28 S
CCTCTTCGGAGGTCCGGTTCGCTGGAAACTGATTTGATCGCAGTGGCTTGAACCGGGCAAAAGTCGTAACAAGGT
AGCCGTAGGTGAACCTGCGGCTGGATCATTATCGAGCTAAGCAAAAAACTCTAGTGCTTCGGCGCTATAGTAGTG
GCAGGAACTATGTGCGGTGCGTTTGCTTGACGTACGACTGCACGCCACAGTGACCCAACGGCGATCGGTTGGTGT
CTGTCCGGTTCTGAGCAGCTGTTGCCTAAGTCCGTGGCTGCGTTGACGAATCGGGGCACGCATACGTCCTGCTAG
TCGGGGTAGCGGATGCGAAGACTCAATGAGCTCCACGCCTGGGAGCTGCCAACCAACCTTTTTTCAACGAATTT
TTCTTAACTAAAGACAAATATCAGTCTTACCGGTGGATCACTCGGTTCGTGGATCGATGAAGAACGCAGCAAACT
GCGATAAGTAGCGTGAATTGCAGACGCTTTGAACGTTAAACTTTCGAACGCACATTGCGCCGTAGGAGTCCTACC
CTGCGGCACATCTGGTTGAGGGTCGTGATCAAAAACAGCAGGACTATGGCTGTAATAGAAATAGCTGGTGAATCA
CGAGTTCGGCCTACCGCCTCTGGCCGAACGTGTTTGCCATAATGCTAGAAGAGGACAATCACTCGGCTCGAGTG
ATTGCCTACCACACGGATGATCTATTAGCTGAAGCAATAGGTCTCGGTCACCGGGTATCATACTAACTATATACT
CTGTGGCGTCTGAGATACAGCTAGTGGGCTTCATACGTGCCTGGTATTGAGAGTATTCGCTCTCCCGACCTCAAC
TCAGGTGTGATTACCCGCTGAACTTAAGCATATCAGTAAGCGAGA

Fig. S1

(A)

GGGTGCCGAAATCGATGGCATACAAAAAGAGGAAGACCAGCGCTTCTTTG 50
ACATGTTCGACCGGGGCAAGAATGGCTACATCATGGCCACCCAAATTGGC 100
ACAATCATGAACGCCATGGAGCAGGACTTTGACGAGAAGACTCTGCGAAA 150
ACTGATCCGCAAATTCGACGCCGACGGCAGTGGCAAGATTGAATTCGATG 200
AGTTCTGCGCACTGGTCTACACCGTGGCCAACACCGTGGACAAGGACACC 250
CTGCGAAAGGAGCTGAAAGAGGCATTCCGTTTATTTGACAAGGAGGGCAA 300
TGGCTACATCTCGCGCCCCACACTCAAGGGCCTTTTGCACGAAATTGCGC 350
CCGACCTCAACGACAAGGACCTGGACGCGGCTGTGGACGAAATTGACGAG 400
GACGGCAGTGGGAAGATTGAGTTCGAAGAGTTCTGGGAACTGATGGCCGG 450
TGACACTGATTAGATGGAAGGATGAATGAATGGGGATGACTGTCAACGAA 500
GATTGTTGGATTGGGGGGAAGAATTTGGGTGAATTCATAAATGGACAATA 550
AACCATG 557

(B)

ATGACCAACTTCGGTACACCACGTAACACCCAACTTCGTATCAAGTCTGA 50
AAACTTGCAGGTAATCCCAGAAGATATTGCCAACCGAACCCATGGTGAAG 100
TTCGTCTCCAATCTGGTACCAATAAATTTGCCTCCCAAAAGTTGATGACC 150
AACTTCGGTACTGGTCGTGATGTGTGCCGTGAGGGAGTCCGTGTCGCTCA 200
GAACCCAGCTGACTTGGCTGAGCTTCCTGAAGAAAAGATCCGTCTTAGTG 250
ATGGTATTGTTCGCCTCCAATCCGGTACCAACAAATATGCCTCACAAAAG 300
GGAATGACAGGCCAGCTTCGGTACCGTCGTGAAACCACCAAAATGATTGA 350
CACCAAGCATCCTGAATACGACCATGAGAAACCCGATCAAGCCGAGATCC 400
CACTTCAATCCGGTACCAATAAGTTTGCTTCGCAGAAGGTAATGATGAGC 450
TTCGGTACCAATCGACGTGAGACCACCAAAATGCTCGATACATCTCACCC 500
TGATTATGATCACACTCAAAGCATTGATCAAACCCAAATTCCATACCAAA 550
TGGGTTCCAATCAATATGCGTCGCAAAAGGGCATGACCAGTTTCGGTCAG 600
CCACGTTGGGAGGTGCTTGACCCCTCAATCAGTTGGCAAAATCGCAAATC 650
TCAAGGCATGGTCCGTCTTCAATCTGGTACCAACAGGTTCGCCTCGCAAG 700
CCGGTTGGTGGGGCTCGTCCGGTTTCGGCACACCCCGCAACGAAGCTGAG 750
GGTGGCGAACTTCCATATGAAGACATGAAGAAGTCTGAATCCATTATCCC 800
ATCACAAGCTGGTTGGAACAAGGGTGATTCCCAGAAAAGGTATGACAGGC 850

TTTGGAATGCCTCGTGA 867

(C)

GAATGGCTGCCGTTGACGTCAACCAACCAATCACCGGACACAAGTCCGCC 50

ATTATCCGCAACTACAATACTAACCCAAGACTCATCTACCAGACCGTATG 100

CGGAGTCAACGGACCACTTGTCATCCTCAACGATGTGAAGTTCCCACAGT 150

TCTCTGAGATCGTCAAGATTACCCTTCCAGACGGCTCAAAGCGTTCCGGA 200

CAGGTTCTTGAAATCTCCAAGAACAAGGCTGTTGTCCAGGTTTTCGAAGG 250

AACCTCCGGAATTGACGCCAAGAACACCATCTGCGAGTTCACTGGAGATA 300

TTTTGCGTACTCCAGTTTCCGAAGACATGCTCGGACGTATCTTCAACGGA 350

TCCGGAAAGCCAATTGACAAGGGACCACCGGTACTTGCCGAGGACTTCTT 400

GGATATCAACGGACAGCCAATCAACCCATGGTCCCGTATCTACCCAGAGG 450

AAATGATCCAGACCGGAATCTCCGCTATCGACGTCATGAACTCTATTGCC 500

CGTGGACAGAAGATTCCAATTTTCTCTGCCTCTGGTCTTCCTCATAACGA 550

GATTGCCGCCCAGATTGTGCGTCAAGGAGGTCTTGTACAGCTTCCAGATC 600

GCCCACACGAGCAGACCAACTTCGCTATCGTCTTTGCCGCTATGGGAGTC 650

AACATGGAGACTGCTCGTTTCTTCAAGCAAGATTTCGAAGAGAACGGTTC 700

CATGGAAAATGTGTGCCTCTTCCTTAACTTGGCCAACGATCCAACCATCG 750

AACGTATCATTACTCCACGTATCGCCTTGACATCCGCTGAATTCCTCGCC 800

TACCAGTGCAAGAAGCACGTGCTTGTTGTCTTGACCGACATGTCCTCATA 850

CGCCGAAGCTCTTCGTGAGGTGTCTGCTGCTCGTGAAGAAGTACCCGGAC 900

GTCGTGGTTTCCCTGGATACATGTACACTGATTTGGCCACCATCTACGAG 950

CGTGCCGGTCGTGTCGAAGGAAGAGACGGATCAATCACACAAATTCCAAT 1000

TCTTACTATGCCAAACGACGATATTACTCACCCTATCCCAGATTTGACTG 1050

GTTACATTACCGAGGGACAGATCTACGTCGATCGTCAGCTTCACAATCGT 1100

CTTATCTACCCACCAATCAACGTACTCCCATCCCTTTCCCGACTTATGAA 1150

GTCTGCTATTGGAGAAGGAATGACAAGAGAAGATCACTCCGATGTGTCTA 1200

ACCAGCTCTACGCTTGTTACGCTATCGGAAAGGACGTGCAAGCTATGAAG 1250

GCCGTCGTCGGAGAAGAAGCCTTGTCATCTGATGATTTGCTCTACCTCGA 1300

GTTCTTGACAAAGTTCGAGAAGAACTTCATCACCCAAGGTCACTACGAAA 1350

ACAGAAGTGTCTTCGAATCCCTCGACATCGGATGGCAACTTCTCCGTATC 1400

TTCCCACGTGAAATGCTCAAGCGTATTCCAGAGTCTACCCTTGAGAAGTA 1450

CTACCCACGTGGAGGAGCCAAGGAATAAGCAGATTTGGAATTGTGTCTTC 1500

ACCCACCTTATCTTGTCTCTTCATTCCATTCCACGCGATACTCTAATTTG 1550

CACTTGACTTTTCTCTCCAAGTCATATTCGTCTTTGTTGTTTCATAACTT 1600

TAGCAGTATAGCTATATATTATATAAGCATGTCCGCCATGCGCAAATCAT 1650

TTTTCAACACCATCACCATCACCACCACTCCACAAAGTGATATTTCATTC 1700

TCGTCATGTAGATATGAAAATTTTTTAATTTTAGTCATTCCAATGTTTCT 1750

TTTTAAACCCCGATTGTTGTAAGTATTCACTTATAAATTGCGATACGCAT 1800

TGCCCCCCGTGATAGCTGGAAATCCAACGTTGTTATTTTTTAAATTCTAA 1850

TGCAATAGTAGGTTATATAGTAACAAGTAAATAAATGGAGCTATCACAAA 1900

AAAA 1904
